# Association of *TNF-α -308G > A* polymorphism with susceptibility to tendinopathy in athletes: a case–control study

**DOI:** 10.1186/s13102-021-00276-2

**Published:** 2021-05-13

**Authors:** Lucas Rafael Lopes, Vitor Almeida Ribeiro de Miranda, João Antonio Matheus Guimarães, Gabriel Garcez de Araujo Souza, Victor Soares Wainchtock, João Alves Grangeiro Neto, Rodrigo de Araújo Goes, Jamila Alessandra Perini

**Affiliations:** 1 Divisão de Pesquisa, Instituto Nacional de Traumatologia e Ortopedia (INTO), Avenida Brasil, 500, RJ zip code 20940-070 Rio de Janeiro, Brazil; 2 Laboratório de Pesquisa de Ciências Farmacêuticas, Centro Universitário Estadual da Zona Oeste (UEZO), Rio de Janeiro, Brazil; 3 Programa de Pós-graduação em Saúde Pública e Meio Ambiente, Escola Nacional de Saúde Pública, Fundação Oswaldo Cruz (Fiocruz), Rio de Janeiro, Brazil; 4 Centro de Trauma do Esporte, Instituto Nacional de Traumatologia e Ortopedia (INTO), Rio de Janeiro, Brazil

**Keywords:** Tendinopathy, Polymorphism, TNF-α, Athletes

## Abstract

**Background:**

High levels of the tumor necrosis factor alpha (TNF-α) induce apoptosis and pro-inflammatory effects for primary degeneration of tendon and development of tendinopathy. The aim of this study was to investigate the association between the *TNF-α* polymorphisms and tendinopathy in athletes.

**Methods:**

Two hundred and seventy athletes (135 tendinopathy cases and 135 controls) were included and genotyped (*TNF-α -1031T > C; -857 C > T; -308G > A*) using TaqMan validated assays. The association of the polymorphisms with tendinopathy was evaluated by a multivariate logistic regression model, using odds ratios (OR) and 95 % confidence intervals (CI).

**Results:**

The variant allele *− 308 A* was significantly associated with patellar (OR: 1.9; 95 % CI: 1.01–3.6) or Achilles tendinopathies (OR: 2.7; 95 % CI: 1.1–6.7). No significant differences were found in allele or genotype distributions of the *− 1031T > C* and *− 857 C > T* polymorphisms between cases and controls. *TNF-α TCA* haplotype was associated with increased tendinopathies risk, either considering all cases (OR: 2.6, 95 % CI: 1.3–5.3), patellar (OR: 3.3, 95 % CI: 1.5–7.3), rotator cuff (OR: 3.1, 95 % CI: 1.4–7.2) or Achilles tendinopathies (OR: 3.8, 95 % CI: 1.1–12.7).

**Conclusions:**

These results suggest that the *TNF-α* polymorphisms could influence the susceptibility to developing tendinopathy among athletes. Knowledge of the *TNF-α* polymorphisms associated to tendinopathy in athletes can further understanding of the inflammatory role in the early stages of the disease and contribute for sports injury surveillance programmes, in which athletes with *TNF-α TCA* haplotype could be early subjected to cryotherapy after training and competition to avoid tendinopathy development.

**Supplementary Information:**

The online version contains supplementary material available at 10.1186/s13102-021-00276-2.

## Background

Tendinopathy is characterized by pain, swelling, structural change and functional limitation of the tendon due to overuse [1, 2]. It is the main reason for clinical musculoskeletal complaint in athletes (15–50 %) [3], which can lead to reduced level of performance or end of one’s sport career [4]. The commonly identified risk factor associated with tendinopathy in athletes are age, sex, metabolic and hormonal concentrations, and high physical load during training and matches according to each sport modality [4, 5].

Single nucleotide polymorphisms (SNPs), a variation of the nucleotide at a single position in DNA sequence, involved with inflammatory process were associated as non-modifiable factors for developing tendinopathies [6–8]. Pro-inflammatory cytokines, such as tumor necrosis factor alpha (TNF-α), and growth factors have been implicated as a mechanism in early stages of tendinopathies [1, 9]. Macrophages, mast cells, fibroblasts and endothelial cells synthesized and released TNF-α cytokine, which is the main chemokine inducer when tendons are mechanically overloaded [1]. TNF-α signaling is mediated by two functionally distinct receptors: TNF-α receptor-1 (TNFR1) and TNF-α receptor-2 (TNFR2). The ligand receptor interaction between TNF-α–TNFR1 is responsible for induce apoptosis and proinflammatory effects, while the interaction between TNF-α–TNFR2 regulates tissue growth and repair [10]. An experimental tendinopathy model produced by overuse shown that *TNF-α* mRNA was increased 11-fold in torn supraspinatus tendon compared to controls [11]. In addition, TNF-α and its receptors were expressed in peritendinous tissue [12], and in rounded/enlarged nucleus human tenocytes, a typical characteristic of tendinopathy [13].

TNF-α is encoded by the gene of the same name, located on chromosome 6p21.3 between the human leukocyte antigen-B (*HLA-B*) and human leukocyte antigen-DR (*HLA-DR*) genes at the major histocompatibility complex class III region [14]. The SNPs in the promoter region of *TNF-α* gene, such as *-1031T > C* (rs1799964), *-857 C > T* (rs1799724) and *− 308G > A* (rs1800629), have shown potential to alters the binding of transcription factors in the DNA, regulating TNF-a expression [15, 16]. The choice of *TNF-α* SNPs was due to their biological relevance in altering the expression of the gene and for have relatively high frequency in different populations [15]. In addition, previous studies associated these *TNF-α* SNPs with some diseases, such as inflammatory bowel diseases [17], Congenital Zika syndrome [18], and cystic fibrosis [19].

We hypothesized that the *TNF-α* SNPs could be associated with a risk of developing tendinopathy in athletes; since high TNF-α expression could be modulated by polymorphisms and TNF-α induce apoptosis and pro-inflammatory effects for primary degeneration of tendon. As far as we know, there are no studies evaluating the influence of *TNF-α* SNPs as possible risk factors involved in the inflammatory molecular mechanism leading to tendinopathy. Thus, this study aimed to investigate the association between the *TNF-α* polymorphisms and tendinopathy in athletes.

## Methods

### Study design and population

This case-control study was approved by the Human Ethics Committee of the *Instituto Nacional de Traumatologia e Ortopedia Jamil Haddad* (protocol number 2.455.630/2017). All participating athletes provided written informed consent and answered a questionnaire about their epidemiological, clinical, sport and training characteristics, as well as tendon injury history and their specific information such as type, location and number of tendinopathy episodes, as previously described [20]. At the end of data collection, a trained observer checked the questionnaire with each athlete, and the database was double-checked by different trained researchers.

The inclusion criteria were Brazilian competitive levels athletes aged 18–45 years old who were recruited between March 2018 and September 2019 at different sports training centres and competitions.

One hundred thirty-five athletes had tendinopathy clinically diagnosed by medical practitioners and confirmed with magnetic resonance image examination (MRI). All tendinopathy diagnoses were confirmed by two blinded specialized orthopaedic surgeons, as described in previous studies [6, 21]. The control group (*N* = 135) consisted of athletes without previous imaging diagnosis of tendinopathy and who were matched with tendinopathy cases for age (difference of ± 2 years), sex and sport modality. The sample size was calculated using Epi Info 7, version 7.1.3. (http://wwwn.cdc.gov/epiinfo/html/downloads.htm) to detect a difference between case and control groups, assuming an odds ratio (OR) of 2.0 with a power of 0.8 and 5 % type I error. The OR was based on previous evidence [22–24], and at least 128 athletes per group was necessary.

### Genotyping of polymorphisms

Genomic DNA was obtained from oral mucosa collected from each athlete by swab. The *TNF-α -1031T > C* (rs1799964), *-857 C > T* (rs1799724) and *− 308G > A* (rs1800629) polymorphisms were genotyped using a TaqMan allelic discrimination assay obtained from Applied Biosystems (C___7514871_10, C__11918223_10 and C___7514879_10, respectively). For all polymorphisms real-time polymerase chain reaction (PCR) reactions were performed on a 7500 Real-Time System (Applied Biosystems, Foster City, CA, USA), and the genotypes were then determined directly. To assure genotyping quality, in each reaction two standardized positive controls of each polymorphism genotype were used.

### Statistical analysis

The normally distribution of studied population was determined by the Shapiro-Wilk test. Comparisons of continuous variables between tendinopathy cases and controls groups were performed using the Student’s t test, and data were presented as mean ± standard deviation (SD). According to distribution and clinical significance the continuous variables (height, age at the beginning of sport practice, years of training and weekly training hours) were divided into quartiles. Categorical data were shown in proportions and differences between the two groups were evaluated using the Chi-squared (χ2) statistic test or Fischer exact test, when applicable.

Deviations from Hardy–Weinberg equilibrium (HWE) were assessed by the goodness-of-fit χ2 test. *TNF-α (-1031T > C, -857 C > T, -308G > A)* allele frequency and genotype distribution were derived by gene counting and frequencies between the two groups were compared using the χ2 test or, when appropriate, the Fisher’s exact test. The haplotype patterns and linkage disequilibrium coefficients (D’ is degree of imbalance in module and R^2^ is degree of correlation) were inferred using Haploview, as previously described [25].

Multivariate logistic regression analyses model were performed to evaluate the possible associations between epidemiological, clinic, sport and training characteristics as much as of the polymorphisms with tendinopathy, which was estimated by the OR with a 95 % confidence interval (95 % CI). As a final regression model used to control possible confounding factors, each variable was introduced considering the biological and statistical significance of the univariate analysis, which a input significance level less than 0.25 (*P* ≤ 0.25) and output significance was 0.05 (*P* ≤ 0.05) at the regression model. The difference was statistically significant when *P* < 0.05. All analyses were performed using the Statistical Package for Social Sciences (SPSS Inc., Chicago, IL, USA, version 20.0).

## Results

**Fig. 1:**
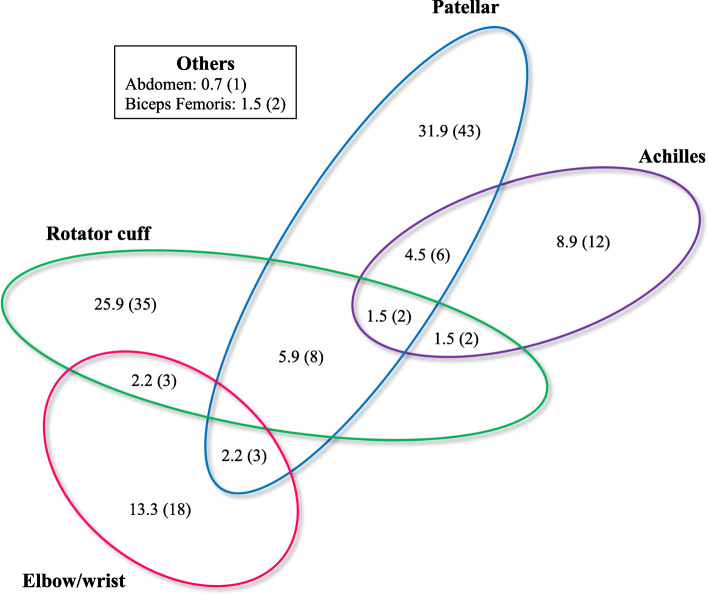
Distribution of the tendinopathy locations in the study athletes (*N* = 135). The values ​​are expressed in % (N)

Of the 135 tendinopathy cases, 24 athletes (17.8 %) reported more than one diseased tendon. The cases reported tendinopathies of the patellar (*N* = 62, 45.9 %), rotator cuff (N = 50, 37.0 %), Achilles (*N* = 22, 16.3 %), wrist (*N* = 15, 11.1 %) and elbow tendinopathy (*N* = 9, 6.7 %) (Fig. 1).

**Fig. 2:**
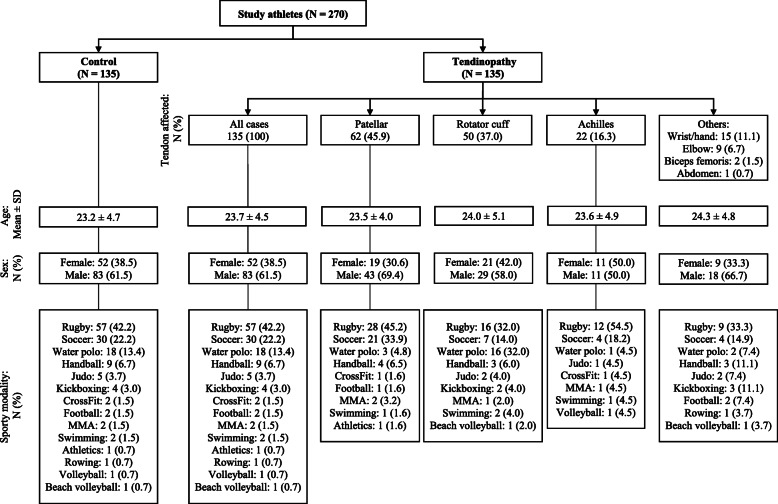
Flowchart of the study population

Age, sex, sport modality of the controls and all cases, as well as cases divided into affected tendon groups, is summarized in Fig. 2. There was no significant difference of the age, sex and sports modality between tendinopathies subgroups (patellar, rotator cuff and Achilles) and control; however, these variables entered the multivariate model for stratified association analyzes, according to the biological importance for the tendinopathy development.

The demographic, clinical, sport and training characteristics variables of all tendinopathy cases and controls were presented in Table 1. In summary, all variables were analyzed to identify possible confounding variables of the true association between SNPs and tendinopathy. Initially, the variables BMI (*P* = 0.09), alcohol consumption (*P* = 0.25), nutritional follow-up by a specialist during a sports career (*P* = 0.002), declared preference member (*P* = 0.21) and weekly training hours (*P* = 0.15) were inserted in the logistic regression model. After multivariate analysis, only BMI and nutritional follow-up remained in this model.

**Table 1:** Epidemiological, clinical, sport and training characteristics of all athlete’s tendinopathy cases and controls (*N* = 270)

Variables	Control (*N* = 135)	Tendinopathy (*N* = 135)	*P-*value^a,b^	Unadjusted OR (CI 95%)	Adjusted OR^b^ (CI 95%)
	N (%)				
Height (centimeters)^d^					
≤ 166	36 (26.9)	34 (25.2)	0.75	1^c^	1^c^
167 – 175	38 (28.4)	40 (29.6)		1.11 (0.58 – 2.13)	0.91 (0.35 – 1.56)
176 – 181	29 (21.6)	25 (18.5)		0.91 (0.45 – 1.86)	0.66 (0.52 – 2.00)
≥ 182	31 (23.1)	36 (26.7)		1.23 (0.63 – 2.40)	0.90 (0.44 – 1.83)
BMI (Kg/m^2^)^e^					
< 25	87 (65.4)	73 (54.5)	0.04	1^c^	1^c^
25 – 29.99	41 (30.8)	49 (36.6)		1.42 (0.35 – 2.39)	1.44 (0.85 – 2.46)
≥ 30	5 (3.8)	12 (8.9)		2.86 (0.93 – 2.50)	3.65 (1.20 – 11.16)
Level of schooling^f^					
Middle school	6 (4.5)	3 (2.2)	0.49	1^c^	1^c^
High school	60 (45.1)	56 (41.5)		1.87 (0.44 – 7.82)	1.80 (0.39 – 8.31)
University education	67 (50.4)	76 (56.3)		2.27 (0.55 – 9.43)	2.19 (0.48 – 10.02)
Alcohol consumption					
No	53 (39.3)	44 (32.6)	0.36	1^c^	1^c^
Yes	82 (60.7)	91 (67.4)		1.38 (0.81 – 2.20)	1.27 (0.76 – 2.14)
Smoking^d^					
No	125 (93.3)	122 (90.4)	0.38	1^c^	1^c^
Yes	9 (6.7)	13 (9.6)		1.48 (0.61 – 3.59)	0.44 (0.57 – 3.59)
Nutritional follow-up					
No	78 (57.8)	52 (38.5)	0.001	1^c^	1^c^
Yes	57 (42.2)	83 (61.5)		2.18 (1.34 – 3.55)	2.31 (1.40 – 3.80)
Side of dominance					
Right	110 (81.5)	104 (77.0)	0.27	1^c^	1^c^
Left	11 (8.1)	20 (14.8)		1.92 (0.88 – 4.21)	1.67 (0.74 – 3.76)
Bilateral	14 (10.4)	11 (8.2)		0.83 (0.36 –1.91)	0.69 (0.29 – 1.63)
Coach^d^					
Certified athletic trainer	92 (68.1)	81 (60.4)	0.54	1^c^	1^c^
Former professional athlete	31 (23.0)	34 (25.4)		1.25 (0.70 – 2.20)	1.20 (0.66 – 2.17)
Both	12 (8.9)	19 (14.2)		1.80 (0.82 – 3.93)	1.53 (0.68 – 3.46)
Age at the beginning of sport practice (years)^d^					
≤ 10	41 (30.3)	41 (30.6)	0.80	1^c^	1^c^
11 – 14	32 (23.7)	29 (21.6)		0.91 (0.47 – 1.76)	0.97 (0.48 – 1.95)
15 – 19	31 (23.0)	37 (27.6)		1.19 (0.63 – 2.27)	1.33 (0.66 – 2.66)
≥ 20	31 (23.0)	27 (20.1)		0.87 (0.44 – 1.71)	0.99 (0.48 – 2.04)
Years of training^d^					
≤ 5	49 (36.3)	38 (28.4)	0.84	1^c^	1^c^
6 – 8	25 (18.5)	27 (20.1)		1.39 (0.70 – 2.77)	1.28 (0.63 – 2.61)
9 – 12	32 (23.7)	35 (26.1)		1.41 (0.74 – 2.67)	1.29 (0.65 – 2.57)
≥ 13	29 (21.5)	34 (25.4)		1,51 (0.79 – 2.90)	1.28 (0.65 – 2.52)
Weekly training hours					
≤ 7	38 (28.1)	32 (23.7)	0.46	1^c^	1^c^
8 – 12	49 (36.3)	38 (28.1)		0.92 (0.49 – 1.73)	0.86 (0.44 – 1.68)
13 – 17	22 (16.3)	24 (17.8)		1.29 (0.61 – 2.73)	1.22 (0.56 – 2.66)
≥ 18	26 (19.3)	41 (30.4)		1.87 (0.95 – 3.70)	1.45 (0.70 – 2.99)

*OR* Odds ratio; *CI* confidence interval. ^a^*P*-value ≤ 0.05 was obtained through the Chi-squared Test (Pearson *p*-value) or Fisher’s exact test. ^b^OR adjusted by BMI and nutritional follow-up. ^c^Reference value. ^d^Information was obtained from 269 athletes. ^e^Information was obtained from 267 athletes. ^f^Information was obtained from 268 athletes.

**Fig. 3:**
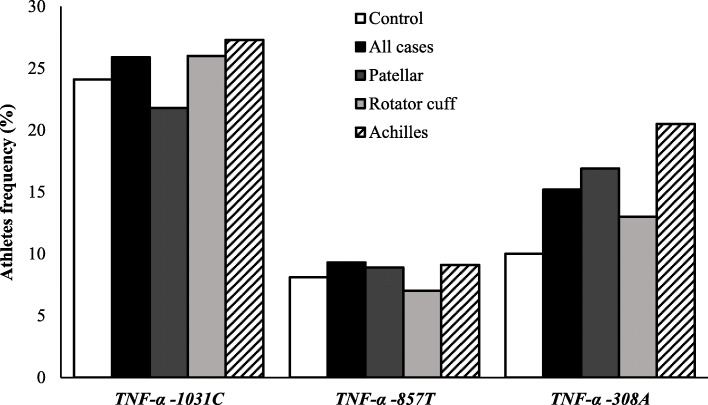
The minor allele frequencies of the SNPs in study population. *P*-value ≤ 0.05 was obtained through the Chi-squared Test (Pearson *P*-value) or Fisher’s exact test

The distribution of *TNF-α* (*-1031T > C, -857 C > T* and *− 308G > A*) SNPs was in Hardy–Weinberg equilibrium. The minor allele frequencies of the *TNF-α* SNPs in the study population are shown in Fig. 3. After adjustment by co-factors of the logistic regression model (age, sex, sport modality, BMI and nutritional follow-up) the *TNF-α-308 A* allele was significantly associated with patellar and Achilles tendinopathies. Moreover, the *TNF-α -308AA* genotype was only present in the tendinopathy cases, either considering all cases, patellar, rotator cuff or Achilles tendinopathies. Considering the recessive co-dominance model (*TNF-α -308GG + GA* versus *AA*) the *TNF-α -308AA* genotype was significantly associated with tendinopathy cases, either considering all cases, patellar and Achilles tendinopathies (Table 2). Despite of the *TNF-α -308AA* genotype suggests a more likely of having tendinopathy nis more than one tendon, when to compared only tendinopathy cases group (one versus 2 or more affected tendons) there was not statistical power due to the decrease of the sample size (data not shown). In addition, no significant differences were found in allele or genotype distributions of the *TNF-α -1031T > C* and *TNF-α -857 C > T* polymorphisms between tendinopathy cases and controls (data not shown).

**Table 2:** Genotypic distributions of the *TNF-α -308 G>A* polymorphism and their association with tendinopathy

*TNF-α* -*308G>A*	Control	Tendinopathy	*P-*value^a^	Adjusted OR (CI 95%)
	**N (%)**			
All tendinopathy cases^b^	***N*** **= 135**	***N*** **= 135**		
*GG*	108 (80.0)	97 (71.9)	0.06	1^d^
*GA*	27 (20.0)	35 (25.9)		1.45 (0.80 – 2.64)
*AA*	0 (0.0)	3 (2.2)		-
*GG+GA*	135 (100.0)	132 (97.8)	0.04	1^d^
*AA*	0 (0.0)	3 (2.2)		-
*G*	243 (90.0)	229 (84.8)	0.007	1^d^
*A*	27 (10.0)	41 (15.2)		1.63 (0.95 – 2.81)
Patellar tendinopathy^c^	***N*** **= 135**	***N*** **= 62**		
*GG*	108 (80.0)	44 (71.0)	0.01	1^d^
*GA*	27 (20.0)	15 (24.2)		-
*AA*	0 (0.0)	3 (4.8)		
*GG+GA*	135 (100.0)	59 (95.2)	0.01	1^d^
*AA*	0 (0.0)	3 (4.8)		-
*G*	243 (90.0)	103 (83.1)	0.04	1^d^
*A*	27 (10.0)	21 (16.9)		1.92 (1.02 – 3.66)
Rotator cuff tendinopathy^c^	***N*** **= 135**	***N*** **= 50**		
*GG*	108 (80.0)	38 (76.0)	0.34	1^d^
*GA*	27 (20.0)	11 (22.0)		1.27 (0.56 – 2.89)
*AA*	0 (0.0)	1 (2.0)		-
*GG+GA*	135 (100.0)	49 (98.0)	0.17	1^d^
*AA*	0 (0.0)	1 (2.0)		-
*G*	243 (90.0)	87 (87.0)	0.35	1^d^
*A*	27 (10.0)	13 (13.0)		1.42 (0.69 – 2.95)
Achilles tendinopathy^c^	***N*** **= 135**	***N*** **= 22**		
*GG*	108 (80.0)	15 (68.2)	0.01	1^d^
*GA*	27 (20.0)	5 (22.7)		1.50 (0.45 – 4.93)
*AA*	0 (0.0)	2 (9.1)		-
*GG+GA*	135 (100.0)	20 (90.9)	0.004	1^d^
*AA*	0 (0.0)	2 (9.1)		-
*G*	243 (90.0)	35 (79.5)	0.03	1^d^
*A*	27 (10.0)	9 (20.5)		2.74 (1.12 – 6.75)

*OR* Odds ratio; *CI* confidence interval. ^a^*P*-value ≤ 0.05 was obtained through the Chi-squared Test (Pearson *P*-value) or Fisher’s exact test to compared control and tendinopathy cases. ^b^ OR adjusted by BMI and nutritional follow-up. ^c^ OR adjusted by Age, sex, sport modality, BMI and nutritional follow-up. ^d^ Reference value.

Seven haplotypes of the *TNF-α (-1031T > C, -857 C > T, -308G > A)* SNPs were inferred, which account 100 % of the study population. The *TCG* haplotype was considered wild-type/reference haplotype due to present the highest frequency in the study population (N = 320, 59.2 %). After adjusting for confounding variables (age, sex, sport modality, BMI and nutritional follow-up), the *TNF-α TCA* haplotype was associated with increased tendinopathies risk, either considering all cases (*P =* 0.006), patellar (*P* = 0.004), rotator cuff (*P* = 0.008) or Achilles tendinopathies (*P* = 0.03) (Table 3).

**Table 3:** Haplotype distributions of *TNF-α* in athletes and their association with tendinopathy

*TNF-α* Haplotypes *– 1031T>C*, *– 857C>T* and *– 308G>A*	Control	Tendinopathy	*P-*value^a^	Adjusted OR (CI 95%)
	**N (%)**			
All tendinopathy cases^b^	***N*** **= 270**	***N*** **= 270**		
*TCG*	172 (63.7)	148 (54.8)	0.11	1^d^
*TCA*	15 (5.6)	31 (11.5)		2.65 (1.32 – 5.30)
*TTG*	14 (5.2)	18 (6.7)		1.74 (0.80 – 3.80)
*TTA*	4 (1.5)	3 (1.1)		0.79 (0.17 – 3.71)
*CCG*	53 (19.5)	59 (21.8)		1.35 (0.86 – 2.11)
*CCA*	8 (3.0)	7 (2.6)		1.03 (0.35 – 2.97)
*CTG*	4 (1.5)	4 (1.5)		1.50 (0.36 – 6.26)
Patellar tendinopathy^c^	***N*** **= 270**	***N*** **= 124**		
*TCG*	172 (63.7)	71 (57.3)	0.10	1^d^
*TCA*	15 (5.6)	17 (13.7)		3.28 (1.47 – 7.31)
*TTG*	14 (5.2)	8 (6.5)		2.09 (0.76 – 5.71)
*TTA*	4 (1.5)	1 (0.8)		0.51 (0.05 – 4.81)
*CCG*	53 (19.5)	22 (17.7)		0.59 (0.59 – 1.98)
*CCA*	8 (3.0)	3 (2.4)		0.25 (0.25 – 3.92)
*CTG*	4 (1.5)	2 (1.6)		0.30 (0.30 – 9.81)
Rotator cuff tendinopathy^c^	***N*** **= 270**	***N*** **= 100**		
*TCG*	172 (63.7)	55 (55.0)	0.01	1^d^
*TCA*	15 (5.6)	13 (13.0)		3.14 (1.36 – 7.24)
*TTG*	14 (5.2)	6 (6.0)		1.88 (0.63 – 5.00)
*TTA*	4 (1.5)	0 (0.0)		-
*CCG*	53 (19.5)	25 (25.0)		1.39 (0.77 – 2.51)
*CCA*	8 (3.0)	0 (0.0)		-
*CTG*	4 (1.5)	1 (1.0)		0.67 (0.07 – 6.32)
Achilles tendinopathy^c^	***N*** **= 270**	***N*** **= 44**		
*TCG*	172 (63.7)	23 (52.3)	0.26	1^d^
*TCA*	15 (5.6)	5 (11.4)		3.79 (1.14 – 12.68)
*TTG*	14 (5.2)	3 (6.8)		1.99 (0.47 – 8.38)
*TTA*	4 (1.5)	1 (2.3)		0.93 (0.09 – 9.90)
*CCG*	53 (19.5)	9 (20.5)		1.33 (0.55 – 3.23)
*CCA*	8 (3.0)	3 (6.8)		3.83 (0.86 – 17.04)
*CTG*	4 (1.5)	0 (0.0)		-

*OR* Odds ratio; *CI* confidence interval. ^a^*P*-value ≤ 0.05 was obtained through the Chi-squared Test (Pearson *P*-value) or Fisher’s exact test. ^b^OR adjusted by BMI and nutritional follow-up. ^c^OR adjusted by Age, sex, sport modality, BMI and nutritional follow-up. ^d^Reference value

## Discussion

Tendinopathy is a serious public health care problem and the knowledge of molecular mechanisms involved in its etiology remains an active area of ongoing research [26]. Any tendon can undergo a tendinopathy process and some modifiable and non-modifiable risk factors are common for the disease in different affected tendon [27]. Recent studies have challenged the “degenerative process” paradigm, suggesting that tendon overload is linked to a complex role of inflammation on tendon homeostasis dysregulation [6, 9, 26, 28]. Although the role of the inflammatory process is not clear, the dysregulation of the proinflammatory cytokines expression and release may contribute to chronic inflammatory responses [9, 26].

Metabolic diseases related to increased adiposity has been identified as important potentially modifiable risk factor for the onset and progression of a variety of tendinopathies [28]. Adipose tissue is tightly associated with tendon inflammation and early tissue degeneration [29]. High BMI and nutritional follow-up by a specialist during a sports career were non-modifiable risk factors for tendinopathy in our athletes. The increased BMI in athletes can result in nutritional monitoring for muscle mass gain, which optimizes the athlete’s performance and physical ability [30]; however, nutritional supplements may be a key component in the etiology of various diseases [31, 32], and diet can contribute negatively with tendon homeostasis [28].

Despite the different risk factors different types of tendinopathies, overloading and mechanical stress may induce the secretion of TNF-α by tenocytes and cause change cellular proliferation, onset of pain and ECM degradation [1]. Under normal physiological conditions TNF-α is not detectable in tendon; however, TNF-α was detect in human tenocytes of Achilles tendinopathy samples, suggesting association with onset tissue apoptosis and in mechanotransduction failure to adapt tendon load [13]. The variation in TNF-α cytokines production is tightly regulated by genetic variants [33]. The present results indicate a positive association between TNF-α *TCA* haplotype and the risk of developing tendinopathy (2-4-fold), which is observed when analyzing only the patellar, rotator cuff or Achilles subgroups. The *TNF-α TCA* (*-1031T > C*, *-857 C > T* and *− 308G > A*) haplotype characterized by the presence of the variant allele of *TNF-α – 308 A*, which promotes loss of transcription factors like activator protein-2 binding, increasing the level of gene transcription [34]. The *TNF-α* SNPs in the promoter region site are strongly in linkage disequilibrium and creates established haplotypes that affect differently gene expression and activity than those of each SNP evaluated separately [33, 34]. This may explain the increased level of *TNF-α* mRNA found in the degenerate tendon [11, 13]; and consequently, contribute to inter-individual variation in tendinopathy development.

Within the Brazilian population the *TNF-α-308AA* genotype is rarer (approximately 0–2 %) [23, 35, 36], and was only observed in the tendinopathy cases (~ 2 %). The total sample size was adequate to detect significant associations with 80 % statistical power; the small number of athletes with different locations of tendinopathies was the main limitation of this study. However, the strength of this study included the control group was matched with all tendinopathy case for age, sex, and sport modality to minimize the influence of the confounding factors. The results can be used to build a database from different populations to identify modifiable and non-modifiable risk factors associated with tendinopathy development in athletes.

It is essential to understand the molecular mechanism involved in the etiology of the disease and for control mechanical stress on the tendon of athletes most likely to develop overuse injuries [37]. The changes in the cytokine production due different genotypes can have significant influence in the tendinopathy, which can impair early tissue regeneration. Identifying genetic changes may improve the prognosis of the disease and clarify new therapeutic targets or personalized training for the athlete, avoiding movement limitations, loss of physical performance and sports ability. Athletes with *TNF-α TCA* haplotype could be early subjected to cryotherapy after training and competition to avoid tendinopathy development. Whole-body cryotherapy decreased serum TNF-α (around 60 %) 24 h following exercise [38]. Thus, this finding can be used in future studies to better understand the influence of genetic factors in the tendinopathy susceptibility and contribute to create sports injury surveillance programmes using genetic information aim reduce cases of the illness in athletes.

## Conclusions

The *TNF-α -308G > A* SNP was potential non-modifiable risk associated with development of disease.

## Supplementary Information

**Additional file 1.**

## Data Availability

Original data are available as Supplementary file 1.

## Abbreviations

95 % CI: : 95 % Confidence interval
χ2: Chi-square
BMI: Body mass index
D’: Degree of imbalance in module
HLA-B: Human leukocyte antigen-B
HLA-DR: Human leukocyte antigen-DR
HWE: Hardy–Weinberg equilibrium
MRI: Magnetic resonance image
mRNA: Messenger RNA
OR: Odds ratio
PCR: Polymerase chain reaction
R²: Degree of correlation
SD: Standard deviation
SNP: Single nucleotide polymorphisms
SPSS: Statistical Package for Social Sciences
TNF-α: Tumor necrosis factor alpha
TNFR1: TNF-α receptor-1
TNFR2: TNF-α receptor-2
